# Patent foramen ovale leading to mismanagement in a mitochondrial myopathy, encephalopathy, lactic acidosis, and stroke‐like episodes patient

**DOI:** 10.1002/ccr3.7723

**Published:** 2023-07-20

**Authors:** Mehri Salari, Masoud Etemadifar, Ronak Rashedi, Romina Rashedi

**Affiliations:** ^1^ Clinical Reasearch Development Unit of Shohada‐e Tajrish Hospital Shahid Beheshti University of Medical Sciences Tehran Iran; ^2^ Functional Neurosurgery Research Center, Shohada Tajrish Neurosurgical Center of Excellence Shahid Beheshti University of medical Sciences Tehran Iran; ^3^ Department of Functional Neurosurgery Medical School, Isfahan University of Medical Sciences Isfahan Iran

**Keywords:** clinical features, MELAS, mitochondrial disease, muscle biopsy

## Abstract

**Key Clinical Message:**

The stroke‐like episodes and brain MRI lesions in MELAS usually have a nonischemic pattern, are resolved over time, and have a migrating pattern that helps us distinguish them from ischemic cerebral infarcts. Nevertheless, conditions such as intracardiac thromboses, PFO, and hypercoagulable state may be present concomitantly, leading to mismanagement. Therefore, further investigation and echocardiography are suggested in MELAS patients.

**Abstract:**

Mitochondrial myopathy, encephalopathy, lactic acidosis, and stroke‐like episodes (MELAS) is the most common maternally‐inherited mitochondrial disorder presenting by stroke‐like episodes, seizures, encephalopathy and muscle weakness. We report the clinical, imaging, echocardiography and muscle biopsy findings of a patient presenting by unique characteristics which have not been reported in previous cases of MELAS. The reported case is a 34 year old man with the history of three times hospitalization due to muscle weakness, encephalopathy, progressive cognitive decline, and gradual visual loss. Muscle biopsy revealed Ragged Red Fibers concomitant with mitochondrial disorders. PFO was found in echocardiography leading to mismanagement of this patient and MR imaging showed ischemic lesions with a progressive pattern. This is the first reported case of MELAS accompanying with PFO. All previous reported cases of MELAS have mentioned a fluctuating characteristic for the ischemic lesions; hence this is the first case of MELAS with the progressive pattern of ischemic lesions.

## Graphical abstract

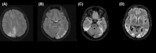

## 1 INTRODUCTION

Mitochondrial disorders consist of a number of diseases, which are mostly due to gene mutations of mitochondrial DNA (mtDNA) or nuclear DNA (nDNA). Clinical traits usually demonstrate as symptoms in high energy‐consuming organs such as the brain, skeletal muscles, myocardium, and endocrine systems.^1^ Mitochondrial myopathy, encephalopathy, lactic acidosis, and stroke‐like episodes (MELAS) is presumably the most common mitochondrial disorder, which is inherited maternally,^2^ and usually becomes symptomatic before the age of 40 years.^3^

MELAS was first introduced in 1984,^4^ which typically presents with stroke‐like episodes, seizures, short stature, encephalopathy, muscle weakness, nausea, vomiting, headaches, diabetes mellitus, exercise intolerance, sensorineural hearing loss, myopathy, lactic acidosis, and RRFs on muscle biopsy.^4^, ^5^, ^6^, ^7^, ^8^ Magnetic Resonance Imaging (MRI), in a company with muscle biopsy and genetic studies, is now the foundation of diagnosis in MELAS cases.^9^

In this case report, we will review the presentation of symptoms, diagnosis challenges and follow‐ups in a patient affected with MELAS. This study has been approved by Shahid Beheshti Medical University research ethics committee and informed consent was obtained from the subject in this study.

## 2 CASE REPORT

A 34‐year old right‐handed man was brought to the emergency department with left upper extremity weakness and significant cognitive decline. He had no significant familial history. In his past medical history, several hospitalizations were found due to similar symptoms.

The patient's first admission dates back to the year 2013, when he was 27 years old, as he was admitted with bilateral visual loss, which had gradually become worsened over 20 days. He also gave a history of new‐onset seizure and was admitted to the neurology ward.

**FIGURE 1:**
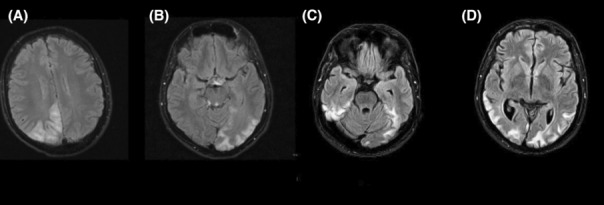
(A) Fluid attenuated inversion recovery (FLAIR) images in the year 2013, when the patient was 27 years old, showing ischemic lesions in the right occipital lobe and posterior parietal regions. (B) FLAIR images in 2014, when the patient was 28 years old, showing left occipital and posterior parietal lobe ischemia. (C, D) In the year 2020, as the patient was 34 years old, FLAIR images revealing bilateral ischemic lesions on the occipitoparietal regions and generalized brain atrophy.

Physical Examination at this time revealed left homonymous hemianopia. Brain Magnetic Resonance Imaging (MRI), Magnetic Resonance Venography (MRV), and Magnetic Resonance Angiography (MRA) was done, and although MRV and MRA study were normal, Brain compound tomography (CT) and MRI findings were suggestive of right temporal lobe ischemia (Figure 1). Laboratory tests that were conducted for the patient were all within normal ranges, including screening for vasculitis. Echocardiography was performed and showed patent foramen ovale (PFO).

The visual field defect was improved gradually, but his seizure‐like movements persisted and were misdiagnosed as pseudo‐seizures.

A year later, in the year 2014, as the patient was 28 years old, he was admitted again because of sudden onset visual loss, which was accompanied by nausea and vomiting. Neuro‐ophthalmological examinations disclosed homonymous hemianopia of the right side. His vision was gradually enhanced over 1 month. Other neurological examinations were unremarkable. Complete paraclinical and laboratory studies were performed, including cerebrospinal fluid analysis, electrocardiogram (ECG), brain CT scan, and brain MRI. All came back negative except the brain MRI which demonstrated signal changes in the occipital lobe (Figure 1). Echocardiography was performed and showed that the left atrial appendage (LAA) was filled with clot, ejection fraction (EF) of 60%, and PFO, and he received warfarin. The patient's strokes were therefore linked to the presence of PFO in this patient.

The last admission was in 2020 when the patient was 37 years old. In this admission, the neurological physical examination revealed left side hemiplegia, and significant cognitive decline (mini mental status examination: 17). Complete laboratory tests, including an autoimmune encephalopathy panel, were done, which were negative. CSF analysis revealed high lactate levels, which along with the high serum lactate level, suggested the diagnosis of mitochondrial encephalopathy, lactic acidosis, and stroke‐like episodes (MELAS) syndrome. Other laboratory tests were normal, which are summarized in Table 1.

**TABLE 1:** Summary of patient's laboratory results.

Laboratory data	Results	Reference range	Unit
Blood tests			
WBC	9200	4.5–11	×10^3^/μL
Hb	12.3	12.6–17.4	g/dl
MCV	87	79–103	fL
Platelets	271,000	140,000–440,000	/uL
ESR	26	0–29	mm/h
PT	13	13±2	s
INR	1.3	1–1.5	
PTT	42	24–39	s^5^
BUN	21	13–43	mg/dL
Cr	1.2	0.5–1.3	mg/dL
Na	136	135–146	mEq/L
K	4.7	3.5–5.5	mEq/L
BS	89	70–100	mg/dL
CPK	184	24–195	U/L
Alk.p	273	64–306	U/L
AST (SGOT)	17	5–33	U/L
ALT (SGPT)	28	Up to 34	U/L
PH (VBG)	7.31	7.3–7.41	
PCO2 (VBG)	57	35–45	mmHg
HCO3 (VBG)	28.5	21–28	mEq/L
PO2 (VBG)	84	80–100	mmHg
Serum lactate	5.7	0.6–2.3	mmol/L
CSF analysis			
RBC	120	0–10	cells/μL
Leukocyte	10	0–5	cells/μL
Neutrophil	8	0%–6% of leukocytes	cells/μL
Glucose	103	40–80	mg/dL
Protein	45	15–45	mg/dL
Lactate	61	10–25	mg/dL

Abbreviation: VBG, venous blood gas.

**FIGURE 2:**
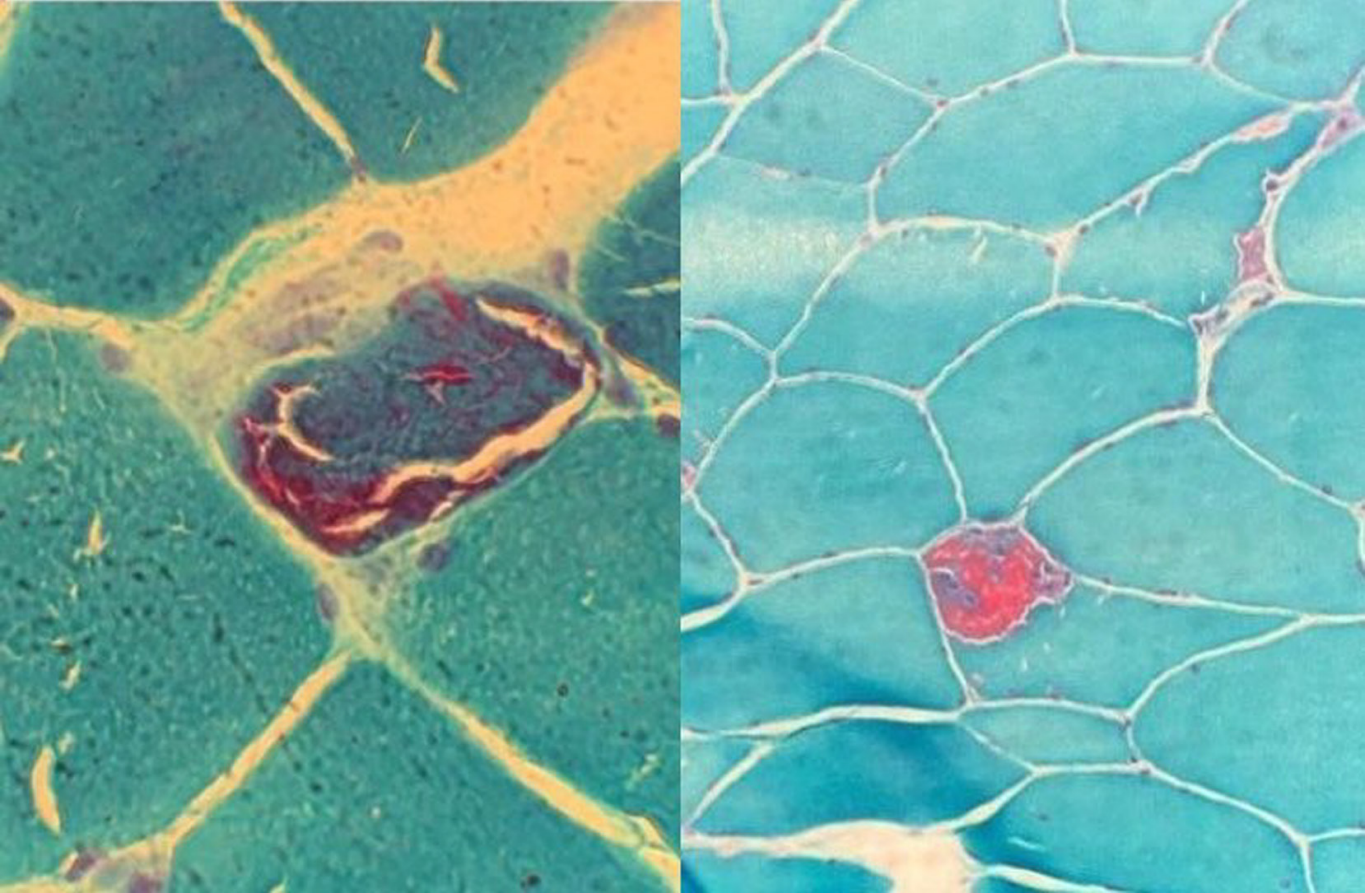
Modified Gomori trichrome section of the biopsy showed increased mitochondrial staining in the subsarcolemmal and intermyofibrillar region of muscle fibers responsible for the Ragged Red appearance.

To confirm the diagnosis of mitochondrial disorders muscle biopsy was performed, was stained with modified Gomori‐trichrome and revealed 10.7% ragged red fibers (RRF) (Figure 2).

## 3 DISCUSSION AND CONCLUSION

Previous studies reporting cases of MELAS have shown specific characteristics in brain MRI imaging of these patients. Transient stroke‐like lesions have been reported in these patients, typically affecting gray matter without being restricted to vascular areas. Involvement of white matter has also been observed in MELAS, being more distinguished in periventricular white matter and centrum semiovale. All the previously reported cases of MELAS have reported a migrating characteristic for the lesions observed in the brain MRI.^10^

The case we report here suffered from progressive cognitive decline, encephalopathy, seizures, and stroke‐like episodes, being compatible with the previously reported cases of MELAS. Nevertheless, this patient had some unique features, never reported in cases of MELAS so far, leading to mismanagement. Since structural heart diseases are more common in young adults with stroke^11^; to rule out the presence of any heart diseases, echocardiography was performed in our patient, and PFO was discovered and confirmed with trans‐esophageal echocardiography (TEE). This finding was first misdiagnosed as the reason for the patient's symptoms, medication was prescribed according to this finding and further investigations were postponed to his next admissions. Despite treatment with anticoagulants, the patient had had another stroke, so the investigation was done again to find a cause that led to a diagnosis of MELAS.

Our literature review revealed no evidence of existing PFO in MELAS cases. Johnson et al. described a MELAS patient in their case report who was once diagnosed with PFO in echocardiography which was later ruled out with TEE. The only reason they could find for the incidence of stroke in their patient was MELAS syndrome.^12^

Another unique presentation in our patient was the pattern of ischemia lesions being different from the previously reported migrating characteristic of the lesions on MRI. Previous studies have shown that the stroke‐like lesions on the brain MRI of MELAS patients tend to resolve over time, and new symptomatic or asymptomatic lesions may appear in new regions of the brain without any signs of the previous lesions.^13^ Nevertheless, lesions had a progressive pattern in this patient, and every time he was admitted, new lesions were added to the old ones without disappearing of previous lesions.

In conclusion, this is the first reported case of MELAS syndrome with PFO and a progressive pattern of ischemic lesions on brain MR imaging. The presence of PFO has led to mismanagement and late diagnosis in this patient, as it may have caused the cerebral infarctions. Nevertheless, although the prevalence is not well known, intracardiac thromboses has previously been reported in cases of MELAS, causing cerebral infarction.^14^ For this reason, further investigations and routine echocardiography are recommended to rule out PFO and intracardiac thromboses in MELAS cases presenting by stroke‐like symptoms.

All procedures performed in this study were in accordance with the ethical standards of Shahid Beheshti Medical University research ethics committee and with the 1964 Helsinki Declarations and its later amendments. The study was approved by the Bioethics Committee of the Medical University of the Medical University of Shahid Beheshti.

## AUTHOR CONTRIBUTIONS

**Mehri Salari:** Conceptualization; project administration; supervision; writing – review and editing. **Masoud Etemadifar:** Supervision; writing – review and editing. **Ronak Rashedi:** Investigation; methodology; writing – original draft. **Romina Rashedi:** Investigation; methodology; writing – original draft.

## FUNDING INFORMATION

This study has not received any financial support from any organization.

## CONFLICT OF INTEREST STATEMENT

The authors declare that they have no conflict of interest.

## ETHICAL STATEMENT

This study has been approved by Shahid Beheshti Medical University research ethics committee and informed consent was obtained from the subject in this study. This study was performed in accordance with the ethical standards as laid down in the 1964 Declaration of Helsinki and its later amendments.

## CONSENT

Written informed consent was obtained from the patient to publish this report in accordance with the journal's patient consent policy.

## Data Availability

The data that support the findings of this study are available from the corresponding author upon reasonable request.
